# The posterior tibial slope does not influence the flexion angle in posterior-stabilized mobile-bearing total knee arthroplasty

**DOI:** 10.1186/s42836-021-00085-5

**Published:** 2021-08-02

**Authors:** Susumu Takemura, Tessyu Ikawa, Yohei Ohyama, Mitsunari Kim, Kunio Takaoka, Yukihide Minoda, Yoshinori Kadoya

**Affiliations:** 1 Department of Orthopaedic Surgery, Osaka City University Graduate School of Medicine, 1-4-3, Asahimachi, Abeno-ku, Osaka, 545-8585 Japan; 2 Department of Orthopaedic Surgery, Saiseikai Nakatsu Hospital Osaka, 2-10-39, Shibata, Kita-ku, Osaka, 530-0012 Japan; 3 Department of Orthopaedic Surgery, Hanwa Joint Reconstruction Center, Hanwa Daini Senboku Hospital, 3176, Fukai Kitamachi, Naka-ku, Sakai, 599-8271 Japan

**Keywords:** Total knee arthroplasty, Tibial slope, Posterior stabilized, Mobile bearing, Flexion angle

## Abstract

**Background:**

It remains uncertain whether an increase in the tibial slope leads to better flexion in posterior-stabilized (PS) total knee prostheses.

**Purpose:**

To compare the intra-operative flexion angle between standard and an additional 10° posterior slope inserts.

**Patients and methods:**

Between December 2014 and February 2015, 22 patients (25 knees) who underwent PS mobile-bearing primary total knee arthroplasty (TKA) were included. Flexion angles were measured using either standard or specially-made inserts. Differences in flexion angles between the two situations were analyzed to determine the relationship between changes in flexion angle and pre-operative flexion angle or body mass index (BMI), and between intra- and post-operative flexion angle.

**Results:**

The difference between the average flexion angle of standard inserts and specially-made inserts was not statistically significant. Although the correlations between changes in flexion angle due to insert difference and flexion angle, pre-operative flexion angle or BMI were not significant, there was a positive correlation between intra-operative and post-operative flexion at 2 years.

**Conclusion:**

The results showed an additional posterior tibial slope by 10° did not affect the intra-operative flexion angle. Surgeons performing PS mobile-bearing TKA do not need to excessively slope the tibial bone cutting to improve the post-operative flexion angle.

**Level of evidence:**

I, Experimental study.

**Supplementary Information:**

The online version contains supplementary material available at 10.1186/s42836-021-00085-5.

## Introduction

The maximum flexion angle after total knee arthroplasty (TKA) is one of the most important factors that determines the post-operative function and patient satisfaction [1, 2]. The post-operative flexion angle is affected by multiple factors, including pre-operative flexion angle, degree of deformity, surgical techniques, and type of prosthesis [3–6].

The posterior slope of the tibia is a possible and long-debated factor that may affect the flexion angle. In cruciate-retaining (CR) prostheses, decreased posterior slope followed by tight flexion gap TKA requires posterior cruciate ligament (PCL) release to increase or maintain the flexion angle [7]. However, the effect of the posterior slope on the posterior-stabilized (PS) prostheses remains controversial [8–15].

We hypothesized that the additional posterior tibial slope would increase the flexion angle of the knee joint even in PS TKA. To test this hypothesis, we created a specially-designed polyethylene insert trial with an additional 10° posterior slope.

The purpose of this study was to compare the intra-operative knee flexion angle between the standard polyethylene insert trial and an additional 10° posterior slope insert trial. This intra-operative measurement enabled us to isolate and evaluate the direct effect of the posterior tibial slope on the flexion angle.

## Patients and methods

This study included 25 knees of 22 consecutive patients with osteoarthritis of the knee (3 males and 19 females; average age, 71.4 years; range, 55–85 years) who underwent primary TKA between December 2014 and February 2015. There were no exclusion criteria. The study was approved by the ethics committee of our hospital. All patients provided informed consent before participation, and all procedures were performed according to the Declaration of Helsinki.

Pre-operative maximum flexion was measured immediately before surgery under general anesthesia. All surgeries were performed by a senior surgeon using a mobile-bearing knee prosthesis (VANGUARD RP High Flex; Biomet Japan, Tokyo, Japan) and according to the following procedure: a straight skin incision was made on the midline of the knee, and the joint was exposed via the medial parapatellar approach. The PCL was resected, and bone resection was performed using the gap balancing technique [16]. Briefly, the bone cut in the proximal tibia was made perpendicular to its long axis using an extramedullary guide. The bone cut in the distal femur was made using a portable navigation system (KneeAlign2; Orth Align Inc., Aliso Viejo, CA, USA) perpendicular to the mechanical axis. After the soft tissue release, the flexion gap was prepared using a gap balancing device (Proflex-G Biomet Japan). All the patellae were resurfaced.

**Fig. 1:**
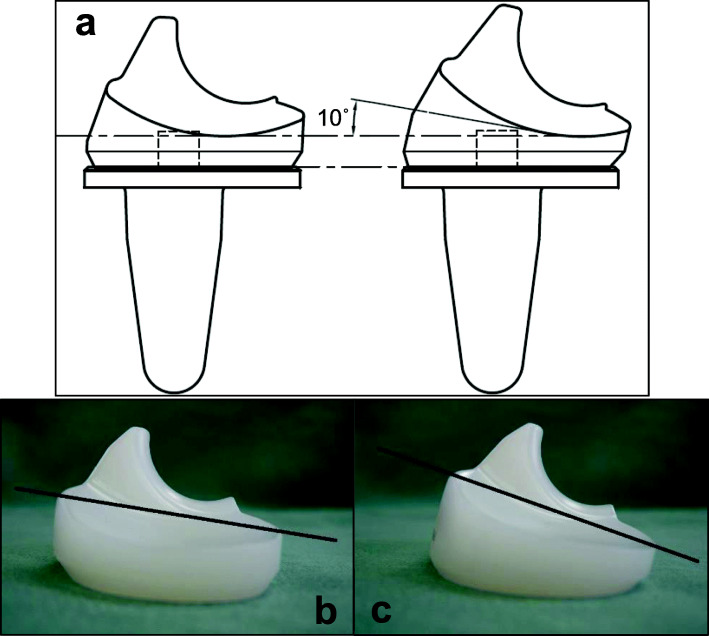
Schematic illustrations and photographs of trial inserts used in the current study. Design image shows the way specially made insert was sloped (**a**). A standard insert (**b**) and a specially-made insert sloped posteriorly by 10 degrees (**c**). A black line is drawn between the anterior and the posterior top of the articular surface

**Fig. 2:**
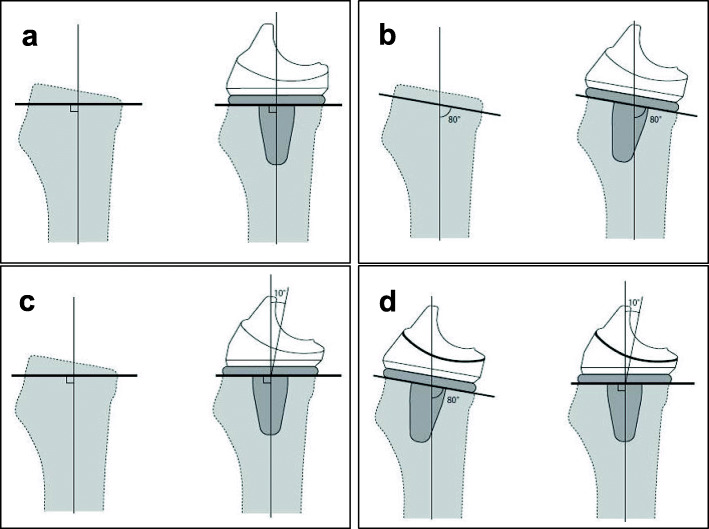
Schematic illustrations of bone cutting (thin lines represent the mechanical axis of the tibia and bold lines represent the surface of bone cutting). Tibial bone resection perpendicular to the mechanical axis with the standard insert (**a**). Tibial bone resection with 10 degrees of the posterior slope with the standard insert (**b**). Tibial bone resection perpendicular to the mechanical axis with the specially-made insert sloped posteriorly by 10 degrees (**c**). Note that the resulting articular surface geometry and the thickness of the thinnest point of the insert are identical between the two inserts (**d**)

After the bone was cut, and the soft tissue was balanced, the trial components were inserted. Intra-operative flexion against gravity was obtained by passively flexing the patient’s hip to 90° and allowing the weight of the lower leg to flex the knee joint, as described previously [17]. The angle of the knee flexion against gravity was measured using a goniometer, as described previously [14]. Two types of polyethylene insert trials were prepared (Fig. 1a): one was the normal polyethylene insert trial (Fig. 1b), while the other had an additional 10° of posterior slope that was specially designed for this study (Fig. 1c). The latter insert was designed to simulate a situation in which the tibial bone was resected at an additional 10° posterior slope. The articular surface geometry and thickness of the thinnest point of the insert were identical as if the posterior slope were increased by 10° during bone cutting (Fig. 2a-d). The order of the two measurements was randomly determined. The soft tissue balance was corrected before the measurements and was not changed between the two measurements. After these measurements, the normal insert was finally implanted.

The patients’ pre-operative flexion angle and body mass index (BMI) were obtained from their medical charts. To determine the correlation between intra-operative and post-operative flexion angle, the post-operative maximum flexion angle was determined in the awake state and supine position at the 2-year follow-up.

### Statistical analysis

All data were analyzed by using SPSS for Windows, version 27 (SPSS Inc., Chicago, IL, USA). The differences in flexion angles against gravity between the two groups were evaluated by using the paired *t*-test. Pearson’s correlation and linear regression analyses were performed to determine the relationship between changes in the flexion angle and other related parameters, including flexion angle with normal insert, pre-operative flexion angle and BMI. The correlation between intra-operative flexion angle and the post-operative flexion angles was also analyzed. Statistical significance was defined as a *P* value less than 0.05. Sample size calculation showed that 21 knees would allow for the detection of a 5° difference (power = 0.8, *α* = 0.05), which was a clinically meaningful difference in knee flexion angle, with a standard deviation of difference of 7.3, as calculated according to the preliminary intra-operative flexion angle under gravity in 25 patients (see ‘Supplementary information’ for more information).

## Results

**Fig. 3:**
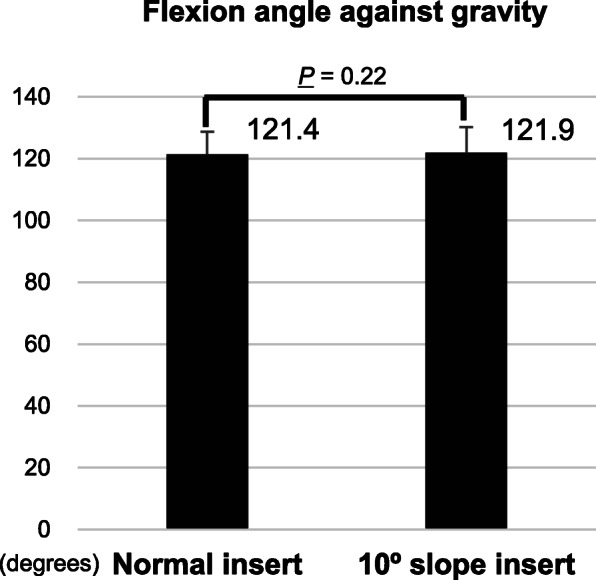
A graph of intra-operative flexion angle against gravity. The graph shows the average intra-operative flexion angles of the standard insert and that of the specially designed 10° posterior slope insert. Values are presented as the means + standard deviations (error bars)

**Fig. 4:**
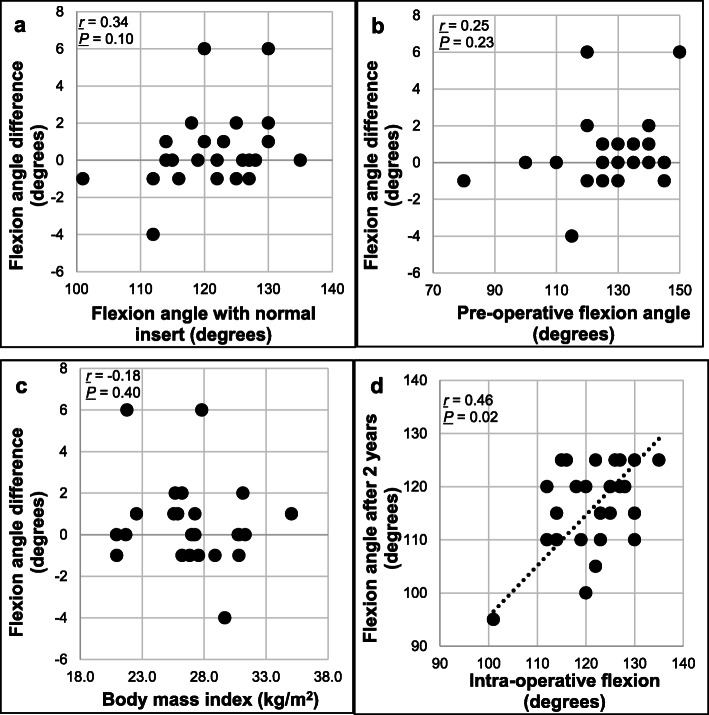
Graphs of the correlation between parameters. The graph shows the correlation between flexion angle with normal insert and intra-operative flexion angle difference (with specially-made insert – with normal insert) (**a**), the correlation between pre-operative flexion angle and intra-operative flexion angle difference (with specially-made insert – with normal insert) (**b**), the correlation between body mass index and intra-operative flexion angle difference (with specially-made insert – with normal insert) (**c**), and the correlation between intra-operative flexion angle and the post-operative flexion angle after 2 years (**d**)

Pre-operative parameters are shown in Table 1. The average intra-operative flexion angle of the standard insert was 121.4° ± 7.4° (mean ± standard deviation), while that of the specially-designed 10° posterior slope insert was 121.9° ± 8.3°. The difference in the flexion angle between the two inserts was not statistically significant (*P* = 0.22, paired *t*-test) (Fig. 3). The average extra flexion angle obtained after increasing the posterior tibial slope angle by 10° was only 0.5° ± 2.0° (range: − 4–6). The flexion angle using the additional 10° posterior slope insert was greater than that using the standard insert in 10 knees, equal in 8 knees and lower in 7 knees. The correlations between change owing to insert difference and flexion angle with normal insert, pre-operative flexion angle or BMI were not significant (*P* > 0.05) (Fig. 4a-c).

**Table 1:** Pre-operative demographic data

Patient characteristics	(*n* = 25)
Age (years)	71.4 ± 7.5
Sex (male / female)	3 / 22
Height (cm)	153.8 ± 5.7
Body weight (kg)	64.1 ± 9.2
BMI (kg/m^2^)	27.1 ± 3.53
Maximum flexion angle in awake state (°)	118.6 ± 15.2
Maximum flexion angle under anesthesia (°)	127.6 ± 15.6

The data are expressed as mean ± standard deviation

The flexion angle 2 years after the operation was 116.2° ± 8.2°. There was a positive correlation between intra-operative flexion angle and post-operative flexion angle (*r* = 0.46, *P* = 0.02) (Fig. 4d).

## Discussion

This was the first study to investigate the impact of posterior slope on flexion angle in the same knee using experimental study protocol. The results of the current study showed that flexion against gravity did not increase when the posterior slope was increased by an additional 10°. Thus, an increase in the posterior slope did not affect the intra-operative flexion against gravity in the model used in this study.

As to an appropriate posterior slope angle, a negative or anterior slope reportedly led to the subsidence of the anterior tibia and dislocation of the insert [18, 19]. In PS TKA, an excessive posterior slope led to anterior post-cam impingement [20]. A recent paper recommended, based on a computer simulation, that the posterior tibial slope should be less than 5° [21]. Furthermore, the study reported that abnormal kinematics, such as anterior sliding of the tibial component and the anterior impingement of the tibial post, were observed when the posterior slope was greater than 5°, arguing that an excessive posterior slope of the tibia in a PS knee should be avoided to prevent damage to the post-cam mechanism.

For PS TKA, some studies investigated the relationship between the posterior tibial slope and the post-operative flexion angle. A previous study reported that the flexion angle improved by 1.8° per degree increase in the posterior slope [15]. Hence, we had expected that the flexion angle would increase by 18° due to the slope being increased by 10°. However, another study reported that there were no differences in the post-operative range of motions between the two groups after using a cutting block with a tilt of 0° and 5°, respectively [12]. Yet another study found that there was no significant difference in the post-operative range of motion between the group with a posterior slope < 10° and the group with a slope ≥ 10° [14]. These studies were of observational nature, and it was not possible to exclude factors affecting the post-operative flexion angle other than the posterior tibial slope.

In this study, the specially-designed 10° posterior slope insert moved the femur more posteriorly than the normal insert. Theoretically, this shift occurred when the bone cut was sloped, and the positive effect increased in the flexion angle because the distance between the posterior surface of the femoral bone and posterior edge of the tibial component increased [4]. The effect of the shift might be obscured by the high conformity of the insert to the femoral component in this study. The prosthesis used in this study had a mobile mechanism and a very conforming upper articulating surface. The rotational freedom probably affected the flexion angle, as high flexion was associated with significant internal rotation of the tibia [22]. Thus, the results obtained in this study may not be directly extrapolated to other PS designs. Further study comparing mobile insert and fixed insert in the same knee will clarify the effect of insert mobility on flexion angle.

In CR TKA, tight flexion gap knee requires PCL release to increase or maintain the flexion angle [7]. The tight PCL causes excessive roll-back of the femur and decreases flexion angle [23]. The most popular method to ease the tightness of the PCL is to increase the posterior slope of tibia. However, the posterior slope itself was reported not to reflect anteroposterior kinematics during deep flexion (90°–120°) [24]. Therefore, the key to deal with tight CR TKA is the adequate tensioning of the PCL and not the increase of the posterior slope.

The merit of this study lies in that the direct effect of the posterior slope of the tibia on the flexion angle was evaluated intra-operatively in the same knee. Two measurements were performed continuously, as the soft tissue balance was corrected before the measurements and remained unchanged between the two measurements. This way, factors other than the insert shape could be completely eliminated, so that the influence on the flexion angle of the tibial slope could be appropriately evaluated.

This study had some limitations. First, the intra-operative flexion angle was examined only against gravity. In agreement with our findings, a previous study reported that there existed a correlation between the flexion against gravity and the post-operative flexion angle when the same implant was used [25]. Therefore, the results of this study also apply to the post-operative flexion angle. Second, the flexion angles were measured manually using a goniometer. Although this is an easy and straightforward method, it often fails to provide accurate and reproducible results. Recently, the standard error of measurement using this device has been reported to be 1.56° (range, 0.52–2.66) [26]. In this study, three markers (the lateral condyle of femur, the head of the fibula, and the lateral malleolus of the foot) were determined and measured. It is unknown whether these markers can be used to reproduce the femoral/tibial axis. However, this measurement evaluates the difference in the flexion angles based on the two types of inserts, and not the flexion angle itself. We believe that the three markers will not detach and will not significantly influence the results of this study. Third, thigh-calf contact, which might limit higher flexion, was not investigated in this study [27]. However, the main conclusion would not be affected by the existence of posterior flesh, which should be identical between the repeated measurements. Finally, this study did not include a large number of samples. However, the sample size was calculated as described in statistical analysis section and 21 knees would allow for the detection of a 5° difference. According to a previous report [15], the flexion angle was expected to increase by 18° due to a 10° slope increase. For these reasons, the number of samples in this study was adequate to serve the purpose of this study.

## Conclusion

This experimental study showed no clinical benefits of flexion angle, even if the tibial slope was increased to 10°. Therefore, in the model used in this study, increasing the tibial slope to increase the flexion angle did not lead to significant improvement. The surgeons performing TKA with the model used in this study needn’t excessively slope the tibial bone cutting to improve the post-operative flexion angle. To determine the generality of these results, further studies using other types of inserts or other models of TKA prostheses are warranted.

## Supplementary Information

**Additional file 1.**

## Data Availability

The datasets generated during and/or analyzed during the current study are available from the corresponding author on reasonable request.

## Abbreviations

PS: Posterior-stabilized
TKA: Total knee arthroplasty
BMI: Body mass index
CR: Cruciate-retaining
PCL: Posterior cruciate ligament
