# Supplementary material for: The posterior tibial slope does not influence the flexion angle in posterior-stabilized mobile-bearing total knee arthroplasty
Source: Arthroplasty. 2021 Aug 2;3:28. doi: 10.1186/s42836-021-00085-5 (PMC8796609; doi:10.1186/s42836-021-00085-5)
Supplement: Supplementary file 1 — Additional file 1. [file 42836_2021_85_MOESM1_ESM.docx]

**Supplementary information**

| Patient  number | Sex | Age at operation  (years) | Height  (cm) | Body weight  (kg) | BMI  (kg/m^2^) | Pre-operative  flexion angle (°) | Post-operative  flexion angle (°) |
| --- | --- | --- | --- | --- | --- | --- | --- |
| 1 | Female | 75 | 154 | 67 | 28.3 | 115 | 114 |
| 2 | Female | 66 | 153 | 51 | 21.8 | 115 | 123 |
| 3 | Female | 71 | 140 | 45 | 23.0 | 125 | 128 |
| 4 | Female | 78 | 148 | 63 | 28.8 | 125 | 116 |
| 5 | Female | 77 | 154 | 60 | 25.3 | 120 | 124 |
| 6 | Female | 71 | 150 | 50 | 22.2 | 130 | 127 |
| 7 | Female | 70 | 158 | 67 | 26.8 | 85 | 105 |
| 8 | Female | 66 | 150 | 66 | 29.1 | 120 | 122 |
| 9 | Female | 83 | 141 | 44 | 21.9 | 120 | 124 |
| 10 | Female | 77 | 148 | 71 | 32.4 | 125 | 114 |
| 11 | Male | 62 | 158 | 56 | 22.4 | 120 | 117 |
| 12 | Female | 80 | 155 | 52 | 21.6 | 100 | 117 |
| 13 | Female | 71 | 153 | 65 | 27.8 | 115 | 115 |
| 14 | Female | 78 | 160 | 60 | 23.4 | 115 | 134 |
| 15 | Female | 77 | 147 | 62 | 28.7 | 120 | 117 |
| 16 | Female | 73 | 160 | 86 | 33.6 | 125 | 120 |
| 17 | Male | 63 | 162 | 60 | 22.9 | 130 | 125 |
| 18 | Female | 72 | 146 | 53 | 24.9 | 140 | 135 |
| 19 | Female | 70 | 153 | 66 | 28.2 | 120 | 120 |
| 20 | Female | 82 | 140 | 40 | 20.4 | 120 | 108 |
| 21 | Female | 80 | 153 | 57 | 24.3 | 135 | 118 |
| 22 | Female | 71 | 160 | 62 | 24.2 | 135 | 131 |
| 23 | Female | 81 | 146 | 40 | 18.8 | 135 | 125 |
| 24 | Female | 70 | 153 | 50 | 21.4 | 145 | 126 |
| 25 | Female | 85 | 150 | 55 | 24.4 | 110 | 118 |
| Average |  | 74.0 | 151.7 | 57.9 | 25.1 | 121.8 | 120.9 |
| Standard deviation |  | 6.2 | 6.2 | 10.5 | 3.7 | 12.5 | 7.3 |
